# Global, regional, and national trends in decubitus ulcer burden from 1990 to 2021 and forecasts to 2040

**DOI:** 10.3389/fpubh.2025.1603321

**Published:** 2025-07-10

**Authors:** Ruijuan Li, Xueneng Yang, Hanbo Chen, Minglin Dong, Jun Shu, Junfei Liu, Ming Zeng

**Affiliations:** The Second Affiliated Hospital of Kunming Medical University, Kunming, China

**Keywords:** decubitus ulcers, Global Burden of Disease, population aging, health inequalities, prevention strategies

## Abstract

**Background:**

Decubitus ulcers, or pressure injuries, are a growing global health issue, particularly among aging populations. However, comprehensive studies on their burden and trends remain limited.

**Methods:**

Using the GBD 2021 database, we analyzed global, regional, and national burdens of decubitus ulcers from 1990 to 2021, including prevalence, incidence, DALYs, and death. Key metrics were assessed through age-standardized rates and absolute counts. Statistical methods such as decomposition analysis and Bayesian age-period-cohort modeling were employed to explore trends and disparities. Future projections were made up to 2040.

**Results:**

While age-standardized prevalence and incidence rates remained stable, DALY and death rates declined globally. Nevertheless, absolute cases, DALYs, and deaths increased significantly, driven by population aging and growth. High-SDI regions showed higher prevalence but lower DALY and death burdens, while low-SDI regions faced severe challenges due to limited resources. Health inequalities persisted, with widening absolute disparities despite narrowing relative inequalities. By 2040, total burden is projected to rise, especially among individuals over 40.

**Conclusion:**

The increasing global burden of decubitus ulcers highlights the need for tailored prevention strategies and resource allocation. These findings offer critical evidence for reducing health disparities and improving global management of this condition.

## 1 Introduction

Decubitus ulcers, also known as pressure injuries, are soft tissue lesions caused by prolonged pressure, leading to ischemic necrosis. They commonly develop over bony areas such as the sacrococcygeal region and heels. Globally, the incidence of these ulcers ranges from 6 to 20% (1, 2). Patients often experience chronic pain, which significantly lowers their quality of life and leads to various physical, psychological, and social challenges (3, 4). These may include social isolation and body image concerns due to wound exudate or odor (3, 4). Moreover, decubitus ulcers present a major economic burden, with daily treatment costs varying from $1.84 to $150. In the United States, annual treatment costs total around $26.8 billion, placing significant pressure on healthcare systems and society (5, 6).

Although previous studies have established a link between decubitus ulcers and chronic diseases or aging (7–9), epidemiological research on this condition is still limited. Most studies focus on specific countries or institutions (2), like nursing homes, with few large-scale global analyses (1, 10). As a result, the changing burden of decubitus ulcers across regions and populations is not well understood. Existing large-scale studies have not fully explored factors such as the sociodemographic index (SDI), health inequalities, population growth, and epidemiological shifts. With the aging population, future trends in the burden of decubitus ulcers remain unclear, making it difficult to improve clinical management and prevention strategies.

This study addresses research gaps by analyzing the global, regional, and national burden of decubitus ulcers from 1990 to 2021, considering factors like age, sex, period, birth cohort, and the SDI. Joinpoint regression was used to track trends, decomposition analysis quantified contributing factors, and correlation analysis highlighted health inequalities. Frontier analysis identified ideal burden levels, while the Bayesian age-period-cohort (BAPC) model predicted future trends up to 2040. These analyses provide comprehensive evidence to guide global health policies, especially in resource-limited areas, helping develop more effective prevention and management strategies to reduce the global impact of decubitus ulcers.

## 2 Materials and methods

### 2.1 Study data and definitions

To examine changes in the burden of decubitus ulcers, we used the GBD 2021 database. In GBD 2021, decubitus ulcers (pressure ulcers or sores) are defined as skin and tissue injuries caused by blood flow obstruction due to prolonged pressure (ICD-10: L89). This study uses prevalence, incidence, deaths, and DALYs as core metrics to measure the burden of decubitus ulcers, focusing on individuals aged 0 to 95 and older. It examines two key indicators: case numbers (raw counts from GBD 2021) and age-standardized rates (per 100,000 population), adjusting for age structure to allow consistent regional comparisons. All data include 95% uncertainty intervals (UI), calculated through aggregation and weighting methods. The SDI, a composite index, correlates positively with per capita income and education years, and negatively with fertility rates among women under 25. GBD 2021 ranks 204 countries and territories into five SDI levels: high, high-middle, middle, low-middle, and low. In this study, SDI is used as a representative measure of economic development, enabling analysis of its impact on the burden of decubitus ulcers.

### 2.2 Statistical analysis

The following statistical methods were used in this study:

(1) Temporal trend analysis: to calculate APC and AAPC, identifying significant trends.
(2) Decomposition analysis: to assess the impact of population age structure, growth, and epidemiological changes on decubitus ulcer burden.
(3) Correlation analysis: to examine the relationship between SDI and age-standardized rates using Pearson correlation coefficients.
(4) Health inequality measurement: using slope index of inequality (SII) for absolute inequality and concentration index (CI) for relative inequality.
(5) Frontier analysis: to analyze the ideal burden of decubitus ulcers based on SDI levels.
(6) Trend projection: trend Projection: Using BAPC model in R to predict future trends from 2022 to 2040.

Statistical analyses and visualizations were performed in R version 4.3.1, with significance set at *p* < 0.05.

## 3 Results

### 3.1 Global level

**Figure 1:**
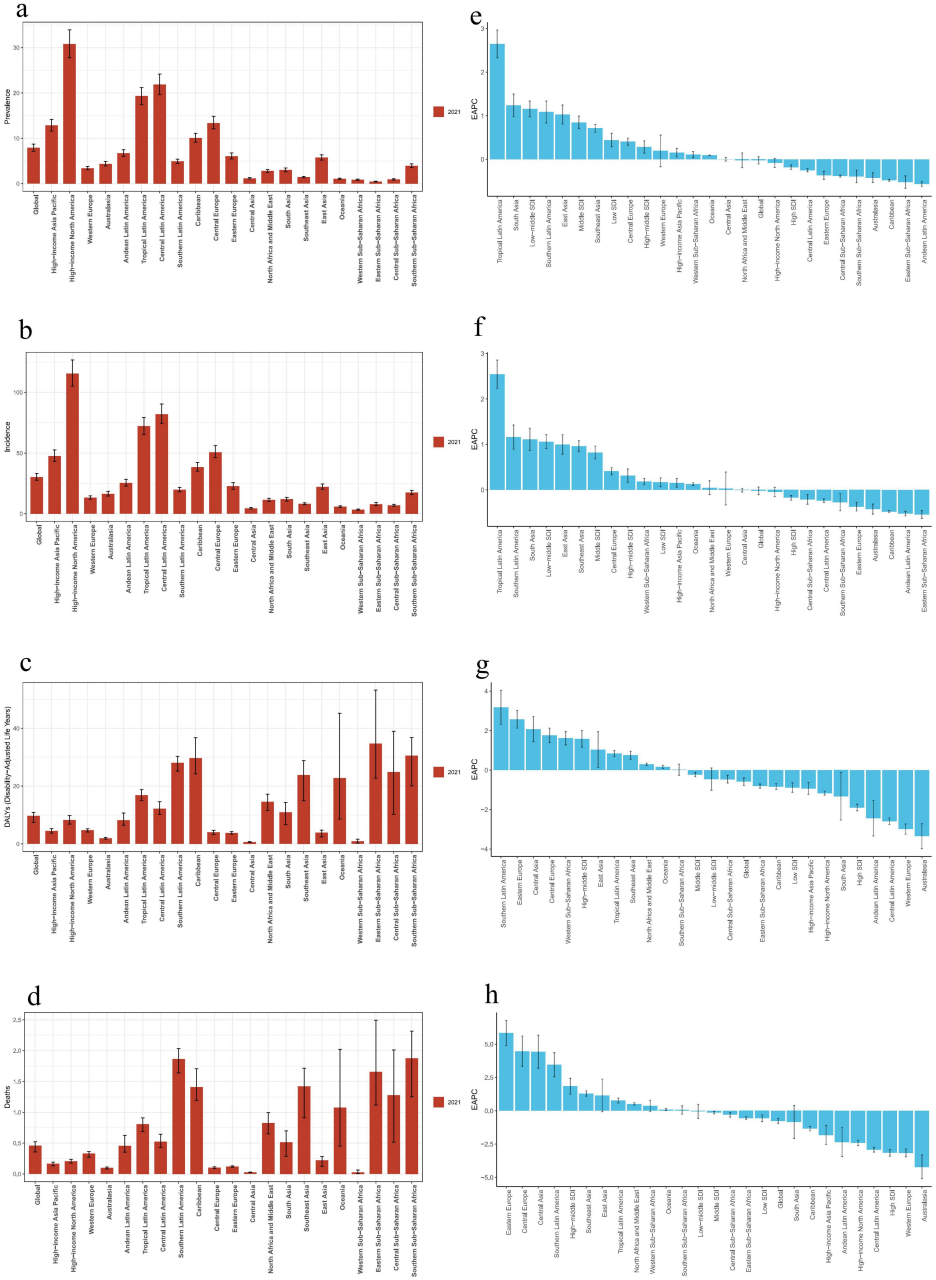
Regional disease burden trends for decubitus ulcers.**(a, e)**: Prevalence burden and EAPC values for 21 regions.**(b, f)**: Incidence burden and EAPC values for 21 regions. **(c, g)**: DALY burden and EAPC values for 21 regions. **(d, h)**: Death burden and EAPC values for 21 regions.) (EAPC, estimated annual percentage change; DALY, disability-adjusted life year).

**Figure 2:**
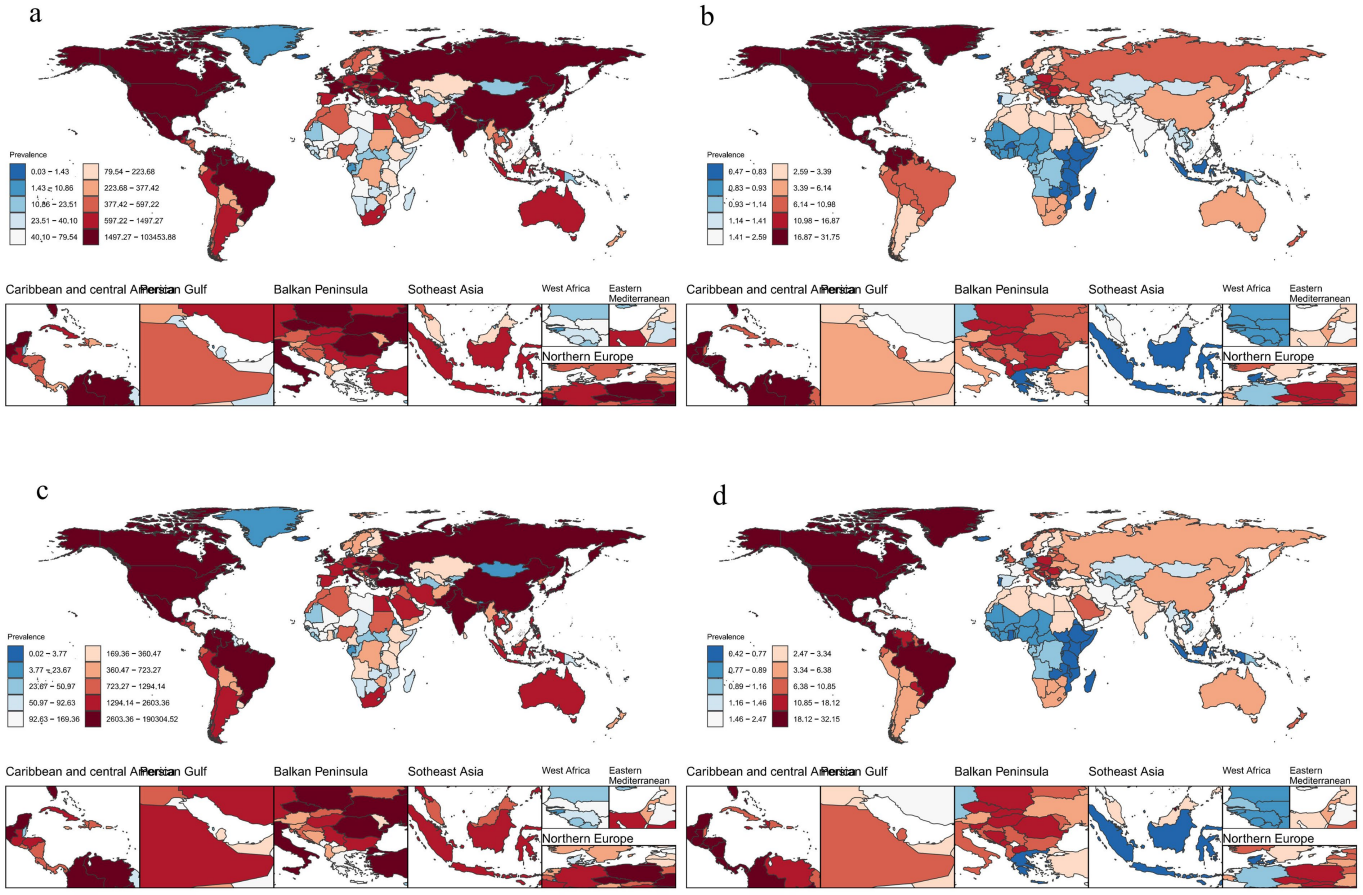
Number of cases and ASPR of decubitus ulcers **(a)**: Number of cases of decubitus ulcers in 1990, **(b)**: ASPR of decubitus ulcers in 1990, **(c)**: Number of cases of decubitus ulcers in 2021, **(d)**: ASPR of decubitus ulcers in 2021. (ASPR, age-standardized prevalence).

In 2021, the global prevalence of decubitus ulcers was 645,588 cases, with an ASPR of 7.9 per 100,000 population, showing no significant change since 1990 (EAPC: −0.0%) (Figures 1e, 2a–d). The number of new cases reached 2,468,318, with an ASIR of 30.3 per 100,000 population, also unchanged (EAPC: −0.0%) (Figure 1F; Supplementary Figures S1a–d). DALYs totaled 803,747 person-years, with an age-standardized rate of 9.7 per 100,000 population, showing a slight decrease (EAPC: −0.6%) (Figure 1G; Supplementary Figures S2a–d). The global death toll was 37,033, with an ASDR of 0.5 per 100,000 population, reflecting a significant decline compared to 1990 (EAPC: −0.8%) (Figure 1H; Supplementary Figures S3a–d; Supplementary Tables S1,S2).

### 3.2 Regional level

In 2021, High-Income North America had the highest ASPR and ASIR of decubitus ulcers, while Eastern and Western Sub-Saharan Africa had the lowest (Figures 1a,b). Eastern Sub-Saharan Africa had the highest age-standardized DALY rate, while Central Asia had the lowest (Figure 1c). The highest ASDR was in Southern Latin America, and the lowest in Central Asia (Figure 1d). From 1990 to 2021, global ASPR and ASIR remained stable, while age-standardized DALY rate and ASDR declined. The largest increases in ASPR and ASIR were in Tropical Latin America, while Andean Latin America saw the biggest decreases (Figures 1e,f). Southern Latin America experienced the largest increase in age-standardized DALY rate, while Australasia saw the greatest decrease (Figure 1g). Eastern Europe had the largest increase in ASDR, while Australasia experienced the most significant reduction (Figure 1h; Supplementary Tables S1, S2).

### 3.3 National level

In 2021, ASPR of decubitus ulcers varied widely across countries, from 0.4 to 31.8 per 100,000 population. The highest rate was in the United States, and the lowest in Iceland (Figure 2d). ASIR ranged from 1.6 to 119.2 per 100,000, with the highest in the United States and the lowest in Iceland (Supplementary Figure S1d). Age-standardized DALY rates varied between 0.2 and 196.4 per 100,000, with Barbados reporting the highest and São Tomé and Príncipe the lowest (Supplementary Figure S2d). Similarly, São Tomé and Príncipe had the lowest ASDR, while Barbados had the highest (Supplementary Figure S3d).

From 1990 to 2021, Brazil saw the largest increase in ASPR, while Portugal experienced the greatest decrease. Malaysia had the highest rise in ASIR, and Portugal the largest decline. Georgia had the highest increase in age-standardized DALY rate, while France showed the greatest decrease. The largest rise in ASDR was in Bulgaria, and the most significant reduction occurred in the United Kingdom (Supplementary Tables S3–S6).

### 3.4 Age and gender trends

**Figure 3:**
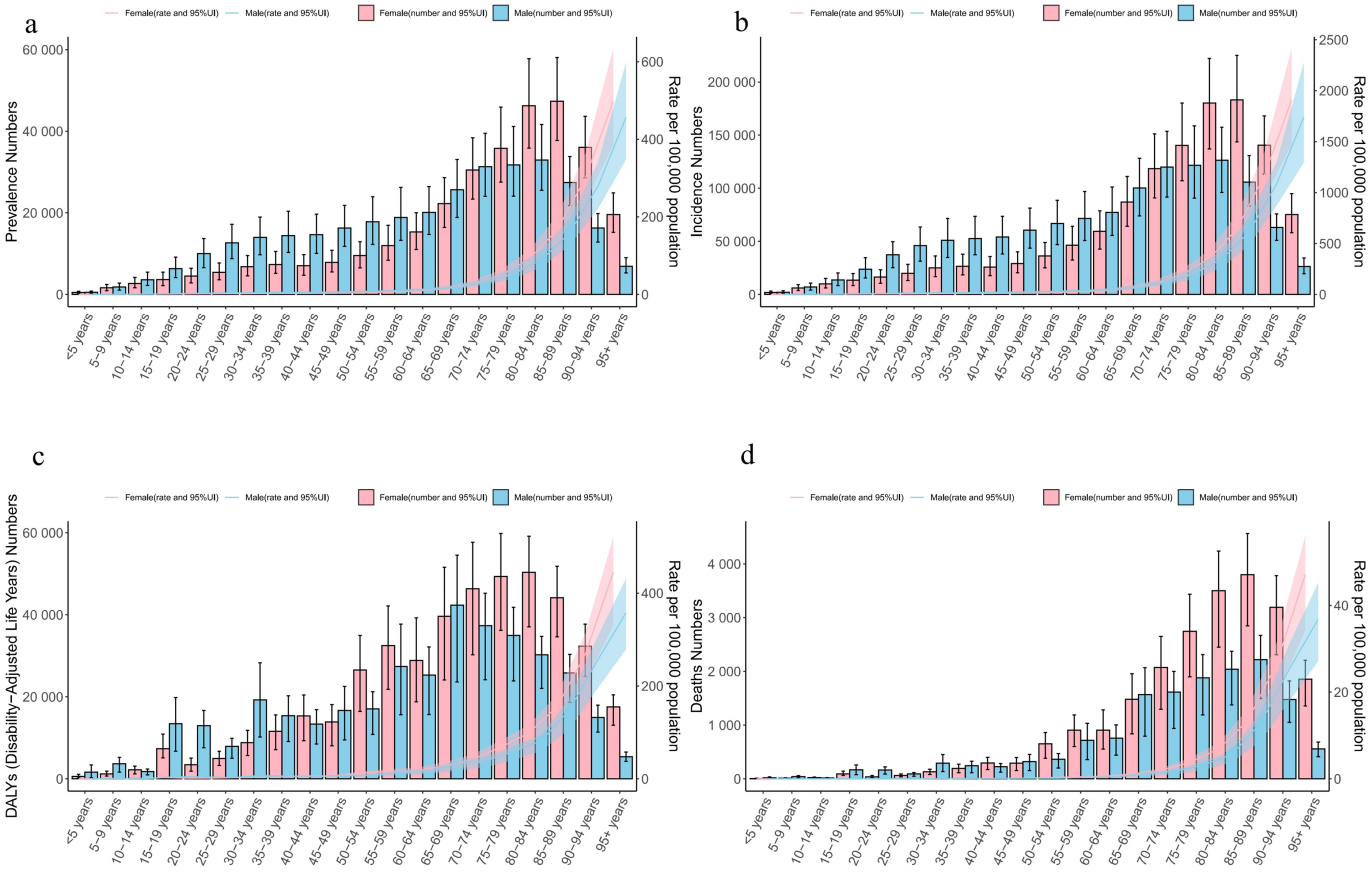
Age and gender differences in global decubitus ulcers in 2021. **(a)**: Gender differences in the number of cases and prevalence rate. **(b)**: Gender differences in the number of new cases and incidence rate. **(c)**: Gender differences in the number of DALYs and DALY rate. **(d)**: Gender differences in the number of deaths and mortality rate. (DALYs, disability-adjusted life years).

In 2021, the global prevalence of decubitus ulcers was similar between sexes but increased with age, peaking in individuals aged 95 and older (Figure 3a; Supplementary Figures S4a,b). Incidence rates were slightly higher in females, with peaks in the older age groups (Figure 3b; Supplementary Figures S4c,d). The DALY rate was higher in females overall, with males peaking at ages 65–69 and females at ages 80–84. Before 50, males accounted for more DALYs, but after 50, females had significantly higher DALYs (Figure 3c; Supplementary Figures S5a,b). Death rates were also higher in females, with the highest rates in those aged 95 and older. The most deaths occurred in the 85–89 age group, and from age 49 onward, mortality was notably higher in females than in males (Figure 3d; Supplementary Figures S5c,d).

### 3.5 Temporal trend analysis

**Figure 4:**
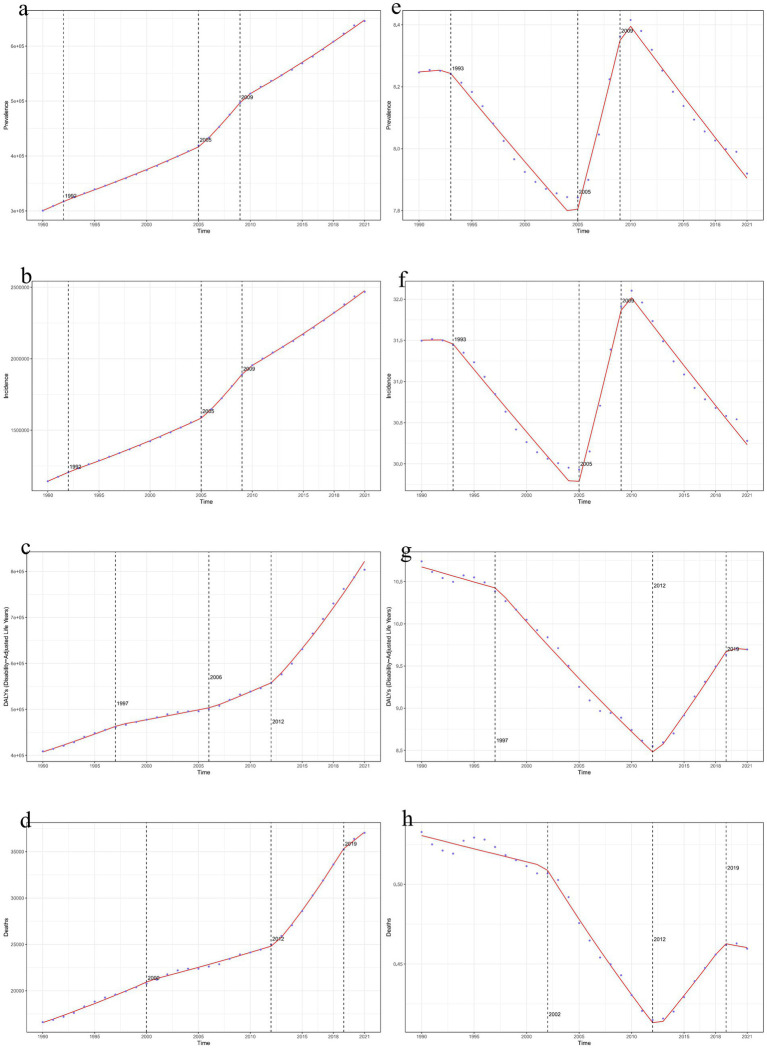
Time trends of global decubitus ulcers in 2021. **(a, e)**: Joinpoint regression model for the number of prevalent cases and ASPR. **(b, f)**: Joinpoint regression model for the number of incident cases and ASIR. **(c, g)**: Joinpoint regression model for the number of DALYs and age-standardized DALY rate. **(d, h)**: Joinpoint regression model for the number of deaths and ASDR. (ASIR, age-standardized incidence rate; ASPR, age-standardized prevalence rate; ASDR, age-standardized death rate; DALY, disability-adjusted life year).

The temporal trend analysis showed an increasing trend in the global number of prevalent cases, incident cases, DALYs, and deaths due to decubitus ulcers (Figures 4a–d). However, the ASPR, ASIR, age-standardized DALY rate, and ASDR all exhibited a declining trend (Figures 4e–h).

### 3.6 Age-period-cohort analysis

The net drift values for global prevalence, incidence, DALY and death rate of decubitus ulcers were −0.06, −0.06, −0.51, and −0.49, respectively, with all metrics peaking among individuals aged 95 and older. Compared to 2004, the relative risks for these indicators peaked in 2009 and 2014. Cohort effect analysis showed a downward trend in prevalence, incidence, DALY and death rate compared to the 1957 birth cohort (Supplementary Figures S6, S7).

### 3.7 Decomposition analysis of decubitus ulcer burden

Decomposition analysis from 1990 to 2021 revealed that population aging and growth were the primary factors driving the increase in prevalent and incident cases of decubitus ulcers. Aging had a negative impact in Eastern Europe but was beneficial in other regions. The drivers of DALYs and death differed by region: in High SDI regions, Western Europe, Central Latin America, and High-Income North America, epidemiological changes were the main negative contributors. In other regions, population aging and growth were the main positive factors. Overall, while the number of prevalent, incident, DALYs, and deaths increased globally, Western Europe and High-Income North America saw declines in DALYs and deaths due to epidemiological changes (Supplementary Figure S8).

### 3.8 Relationship between decubitus ulcer burden and SDI

**Figure 5:**
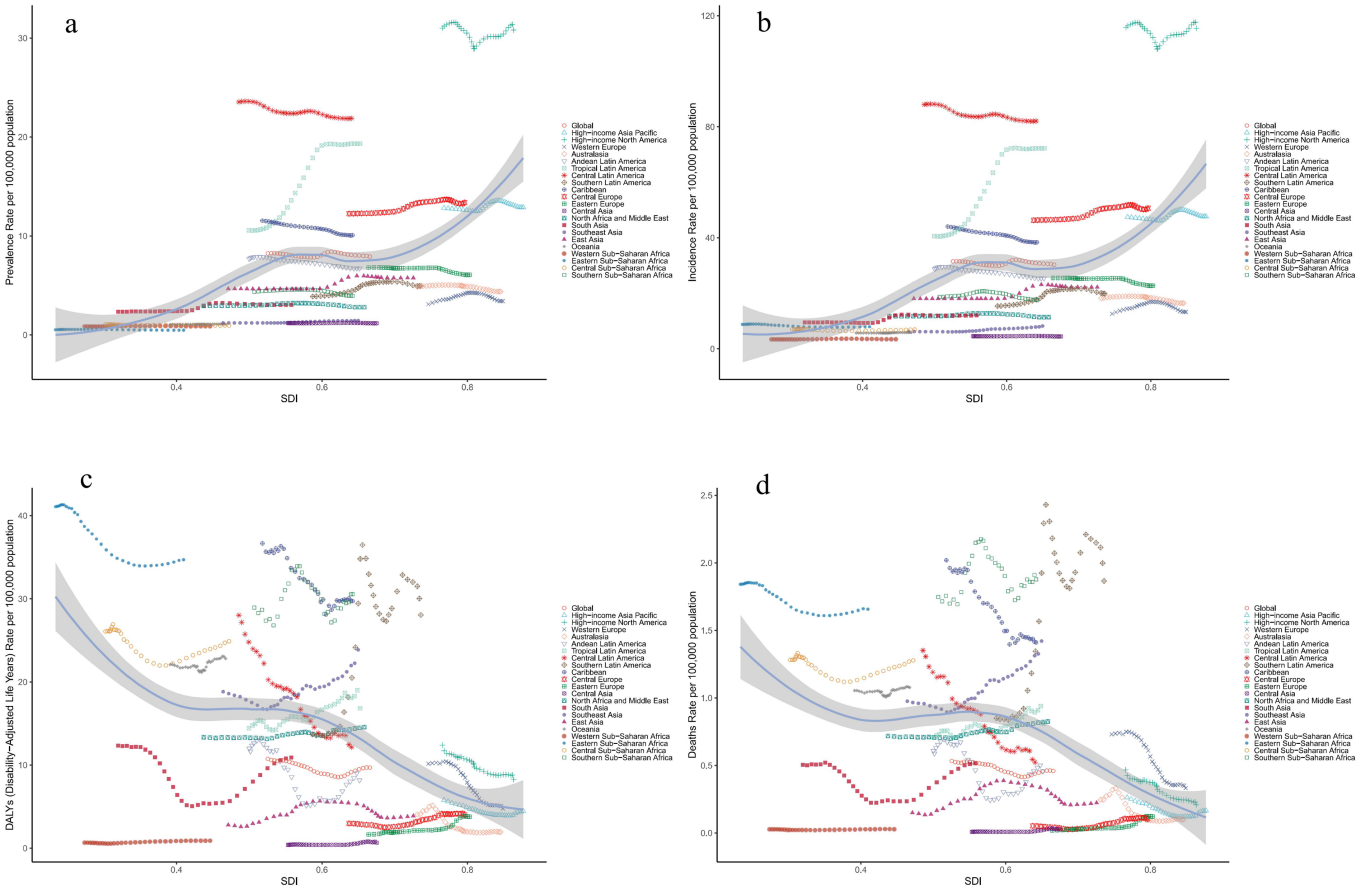
Relationship between the disease burden of decubitus ulcers and SDI in 2021 globally and across 21 regions. **(a)**: ASPR **(b)**: ASIR **(c)**: Age-standardized DALY rate. **(d)**: ASDR. (ASIR, age-standardized incidence rate; ASPR, age-standardized prevalence rate; ASDR, age-standardized death rate; DALY, disability-adjusted life year; SDI, socio-demographic index).

Pearson correlation analysis revealed that SDI was positively correlated with the ASPR(*r* = 0.6077, *p* < 0.01) and ASIR (*r* = 0.5475, *p* < 0.01), indicating higher rates in regions with higher SDI (Figures 5a,b). In contrast, age-standardized DALY rate (*r* = −0.4001, *p* < 0.01) and ASDR (*r* = −0.3706, *p* < 0.01) were negatively correlated with SDI, suggesting lower age-standardized DALY rate and ASDR in high-SDI regions (Figures 5c,d).

Over the past 30 years, the ASPR and ASIR of decubitus ulcers generally matched expectations based on SDI levels. Most regions had burdens below expectations, with exceptions like High-Income North America, which had higher-than-expected burdens. Some developing countries, such as Haiti, also showed higher burdens, while certain high-income countries, like San Marino, had lower-than-expected burdens (Supplementary Figures S9a,b). Globally, DALY burdens were mostly lower than expected, though regions like Oceania had higher-than-expected burdens. Some developing countries, such as Guinea, reported lower-than-expected DALY burdens, while high-income countries, like the Bahamas, exceeded expectations. Death burdens were generally lower than expected, but regions such as the Caribbean had higher-than-expected mortality, while countries like Sierra Leone reported lower-than-expected death (Supplementary Figures S9c, d).

Furthermore, no significant correlation was observed between baseline ASPR, ASIR, age-standardized DALY rate, or ASDR in 1990 and their respective EAPC values, suggesting that baseline burden levels did not strongly influence trends in ASR changes. Similarly, no significant association was found between SDI and EAPC values for ASPR, ASIR, or ASDR. However, there was a significant negative correlation between SDI and the EAPC of the age-standardized DALY rate, indicating that countries with higher SDI were more likely to exhibit a declining trend in DALY rates by 2021(Supplementary Figure S10).

### 3.9 Health inequality analysis

Based on the correlation between decubitus ulcer burden and SDI, this study analyzed health inequality trends from 1990 to 2021. The SII for prevalence increased from 5.94 to 8.93, while the CI decreased from 0.42 to 0.39 (Supplementary Figures S11a,e). For incidence, SII rose from 22.17 to 34.43, with CI declining from 0.41 to 0.38 (Supplementary Figures S11b,f). The SII for DALYs increased from 0.74 to 2.88, while CI shifted from 0.02 to −0.02 (Supplementary Figures S11c,g). For death, SII rose from 0.17 to 0.35, and CI dropped from 0.16 to 0.04 (Supplementary Figures S11d,h).

### 3.10 Frontier analysis of ideal decubitus ulcer burden and SDI association

Frontier analysis explored the ideal disease burden scenario for countries based on specific SDI conditions. Results identified five low-SDI countries closest to the frontier fit line (marked in blue) and five high-SDI countries furthest from the frontier fit line (marked in red). The 15 countries furthest from the frontier fit line across all SDI levels were marked in black. For prevalence and incidence burdens, the countries furthest from the frontier fit line were the United States, China, India, Brazil, and Japan (Supplementary Figures S12a–d). For DALY burden, the furthest countries were India, China, the United States, Brazil, and Thailand. Regarding mortality burden, the countries furthest from the frontier fit line were India, China, Brazil, Thailand, and the United States (Supplementary Figures S12e–h).

### 3.11 Forecasting the burden of decubitus ulcers

Using population projection data for 2022–2040 and the Bayesian age-period-cohort (BAPC) model, this study predicts trends in the burden of decubitus ulcers over the next 20 years. Firstly, the age-standardized prevalence rate is expected to remain stable, while the total number of prevalent cases will increase. Prevalence rates are projected to rise in individuals aged 40–54 and those over 80, while declining in those under 35. Secondly, the age-standardized incidence rate is anticipated to remain stable, with an increase in the total number of incident cases. Incidence rates are expected to increase in individuals aged over 40 and decline in those under 35. Thirdly, the age-standardized DALY rate is projected to decline, although the total number of DALYs will increase. DALY rates are expected to rise in the 55–84 age group and decrease in other age groups. The total DALYs will increase for individuals over 40 years and decrease for those under 40. Finally, while the age-standardized death rate is expected to remain stable, the total number of deaths will increase. Mortality rates are projected to rise in the 55–84 age group and decline in other age groups. The number of deaths is expected to increase in individuals over 40 years and decrease in those under 40 (Supplementary Figures S13–S15).

## 4 Discussion

### 4.1 Overview of global trends and key findings

This study examined trends and factors influencing the global, regional, and national burden of decubitus ulcers from 1990 to 2021. While global ASPR and ASIR remained stable, age-standardized DALY rates and ASDR declined, the total number of cases, DALYs, and deaths continued to rise, with notable regional disparities. The burden was higher among females and older populations. High-SDI regions had higher prevalence and incidence rates but lower death and DALY rates. Population aging and growth were identified as key drivers of the increasing burden. Despite narrowing health inequalities, disparities persist. Projections suggest the burden will continue to rise, especially among older age groups, emphasizing the need for targeted prevention strategies and better resource allocation.

### 4.2 Research innovation

This study analyzed the decubitus ulcer burden from 1990 to 2021, evaluating key indicators (such as ASPR, ASIR), age-standardized DALYs, and ASDR at global, regional, and national levels. Using methods like temporal trend analysis, APC analysis, and decomposition analysis, it explored the impacts of population aging, growth, epidemiological changes, and SDI on the burden. Health inequality and frontier analyses identified regions with significant disparities and countries needing priority interventions. Future trends were projected, providing insights for public health policy and resource allocation, thus deepening the understanding of decubitus ulcer burden.

### 4.3 Risk factors and demographic influences

Studies have shown that decubitus ulcer occurrence is closely linked to various physiological and social factors. Consistent with previous research (11), this study found a steady increase in decubitus ulcer cases over the past 30 years, with older adults identified as high-risk. Contributing factors include reduced skin elasticity, poor circulation, limited mobility (12), care quality (13, 14), nutrition, and medical equipment use (15–18). While some studies suggest that male sex is a risk factor (19, 20), our findings did not support this, possibly due to differences in data sources (21). The burden is notably higher among the older adult, likely due to limited mobility, chronic conditions, and malnutrition (15–17). Advances in medical care, such as platelet-rich plasma therapy (13, 14, 18, 22) and alternating pressure air mattresses (18, 23, 24), have proven effective in reducing decubitus ulcer incidence, which aligns with the findings from the APC model in this study. In conclusion, decubitus ulcers are influenced by multiple factors, including age, sex, and healthcare context. Future interventions should focus on individualized strategies, especially for high-risk populations (7).

### 4.4 Regional disparities and the role of SDI

The prevalence of decubitus ulcers is closely linked to regional economic levels, with significant differences in disease burden across regions with varying SDI levels (25). High and upper-middle SDI regions, such as North America, have higher ASPR and ASIR, while regions with lower SDI, like Sub-Saharan Africa, show lower rates. In high-SDI regions, abundant medical resources help reduce death and DALY burdens, despite higher ASIR, which aligns with previous studies (26–28). In contrast, the higher burden in low-SDI regions reflects the negative impact of limited healthcare resources. The study also found declining age-standardized DALY and ASDR trends in high-income countries, highlighting the effectiveness of preventive care in controlling decubitus ulcers (29).

### 4.5 Drivers of disease burden: aging and epidemiological shifts

Decomposition analysis showed that the increasing burden of decubitus ulcers is mainly driven by population aging and growth. From 1990 to 2021, aging significantly contributed to the rise in prevalent and incident cases. However, the relatively slow increase in death rates and DALYs indicates that, while aging is a major factor, epidemiological changes, particularly in high-income countries, have also played an important role.

### 4.6 Frontier analysis and policy implications

Frontier analysis provides valuable insights for preventing and managing decubitus ulcers. In low-SDI countries like Niger, where the burden is near the frontier, resources can be redirected to address more urgent health issues. In high-SDI countries like Japan, where the burden is significantly below the frontier, more investment in healthcare and preventive measures is needed. For countries with the largest gaps from the frontier, such as China and the United States, prioritizing decubitus ulcer management is essential regardless of SDI level (30). Tailored policies should be developed based on SDI levels to optimize resource allocation and effectively address the disease burden.

### 4.7 Health inequality trends

Over the past three decades, health inequality in the burden of decubitus ulcers has remained stable. An increase in the slope index shows widening absolute disparities globally, while a decrease in the concentration index indicates a reduced relative impact of economic inequality. Specifically, low-income countries bear a heavier burden, especially in DALYs and death.

### 4.8 Future projections and public health recommendations

Projections from this study suggest a significant increase in the burden of decubitus ulcers by 2040. Health management should focus on individuals over 40, particularly women, with proactive prevention and treatment strategies. Specific interventions include: establishing risk group profiles and conducting regular follow-ups in communities; promoting “turning schedules” and using pressure-relieving cushions in healthcare institutions; and launching a “Decubitus Ulcer Prevention Awareness Month” in the media to raise public awareness. These measures can effectively reduce the burden of decubitus ulcers and improve overall health.

## 5 Conclusion

This study highlights global trends in the burden of decubitus ulcers, emphasizing regional and gender disparities as well as health inequalities. While global ASDR and age-standardized DALY rate have generally declined, the burden remains higher than expected in some high-income countries. With population aging, the burden is likely to increase, particularly among older women. The study calls for tailored prevention and treatment strategies based on regional characteristics, providing scientific evidence to inform public health policies and health management in aging societies.

## 6 Limitation

This study, based on GBD 2021 data and the BAPC model, analyzes the global, regional, and national burden of decubitus ulcers. However, it has several limitations. First, data quality varies across countries, especially in low- and middle-income regions, where data gaps may lead to underestimation of the burden. Efforts should be made to strengthen data collection in these regions to improve the accuracy of burden estimates. Second, individual risk factors such as diabetes, obesity, chronic diseases, and malnutrition were not considered, which limits a more nuanced understanding of the mechanisms behind decubitus ulcer occurrence. Future studies could incorporate these factors to provide a more detailed explanatory framework. Third, trend projections are based on historical data and may not fully reflect the potential impacts of healthcare policies, technological advancements, or socioeconomic changes. Incorporating these elements could enhance the reliability of future forecasts. Fourth, the possible association of chronic inflammation during the disease and the role of anti-inflammatory drugs depending on gender were not explored, which may contribute to an incomplete understanding of gender-specific risks and therapeutic responses. Finally, the study focuses on macro-level trends and does not address individual-level care variations and specific intervention strategies, which may limit the applicability of the findings to clinical practice.

## 7 Implications for clinical practice

This study clarifies gender and age differences in pressure injuries, quantifies the current burden, evaluates prevention and care effectiveness, and predicts future trends. It highlights future treatment needs, identifies health inequalities, and supports equitable resource allocation, ultimately providing actionable strategies based on the data.

## Data Availability

The raw data supporting the conclusions of this article will be made available by the authors, without undue reservation.
