# Supplementary figures and images for: Global, regional, and national trends in decubitus ulcer burden from 1990 to 2021 and forecasts to 2040
Source: Front Public Health. 2025 Jul 10;13:1603321. doi: 10.3389/fpubh.2025.1603321 (PMC12287041; doi:10.3389/fpubh.2025.1603321)

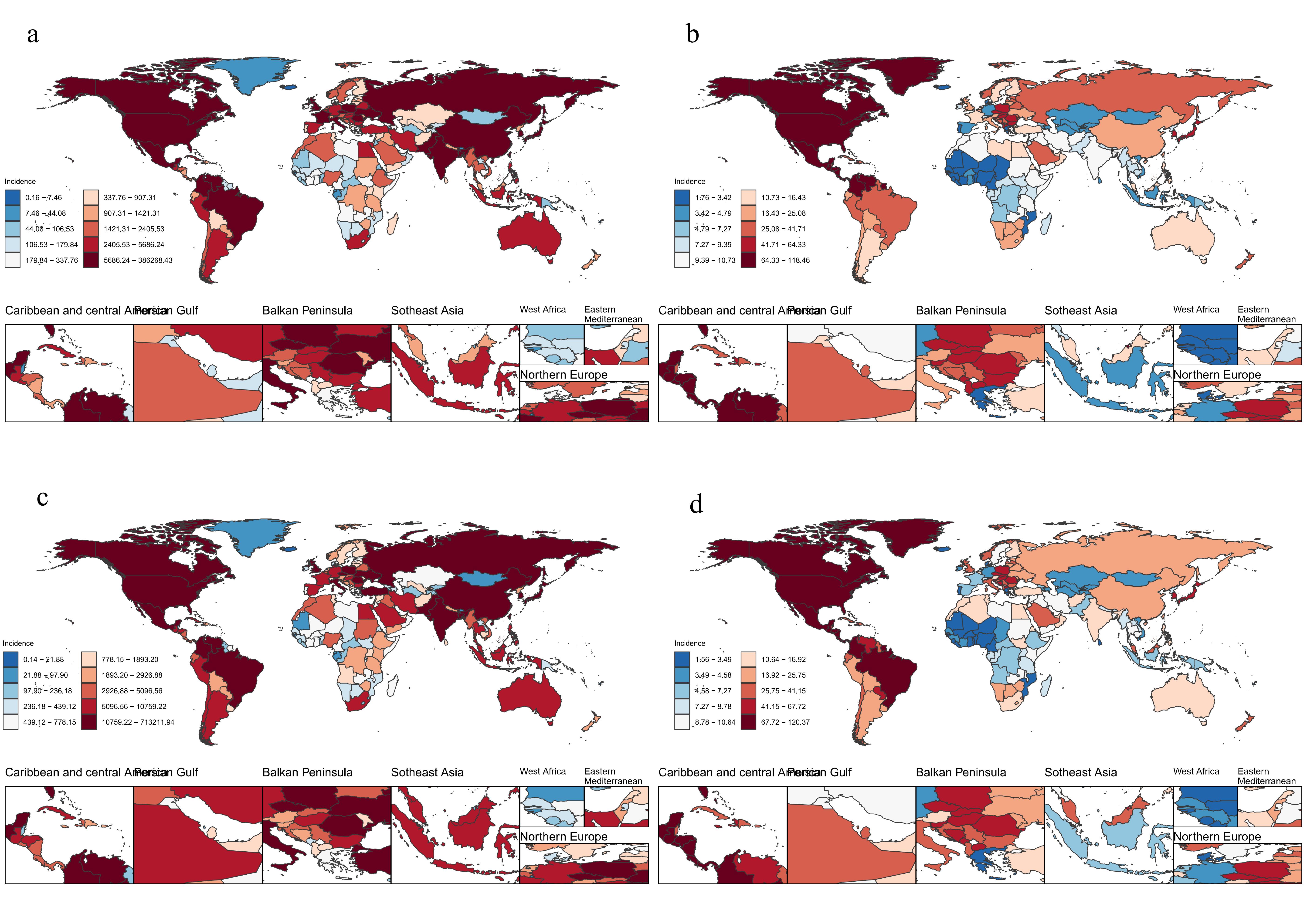

Supplement: Supplementary file 1 [file Image_1.jpeg]

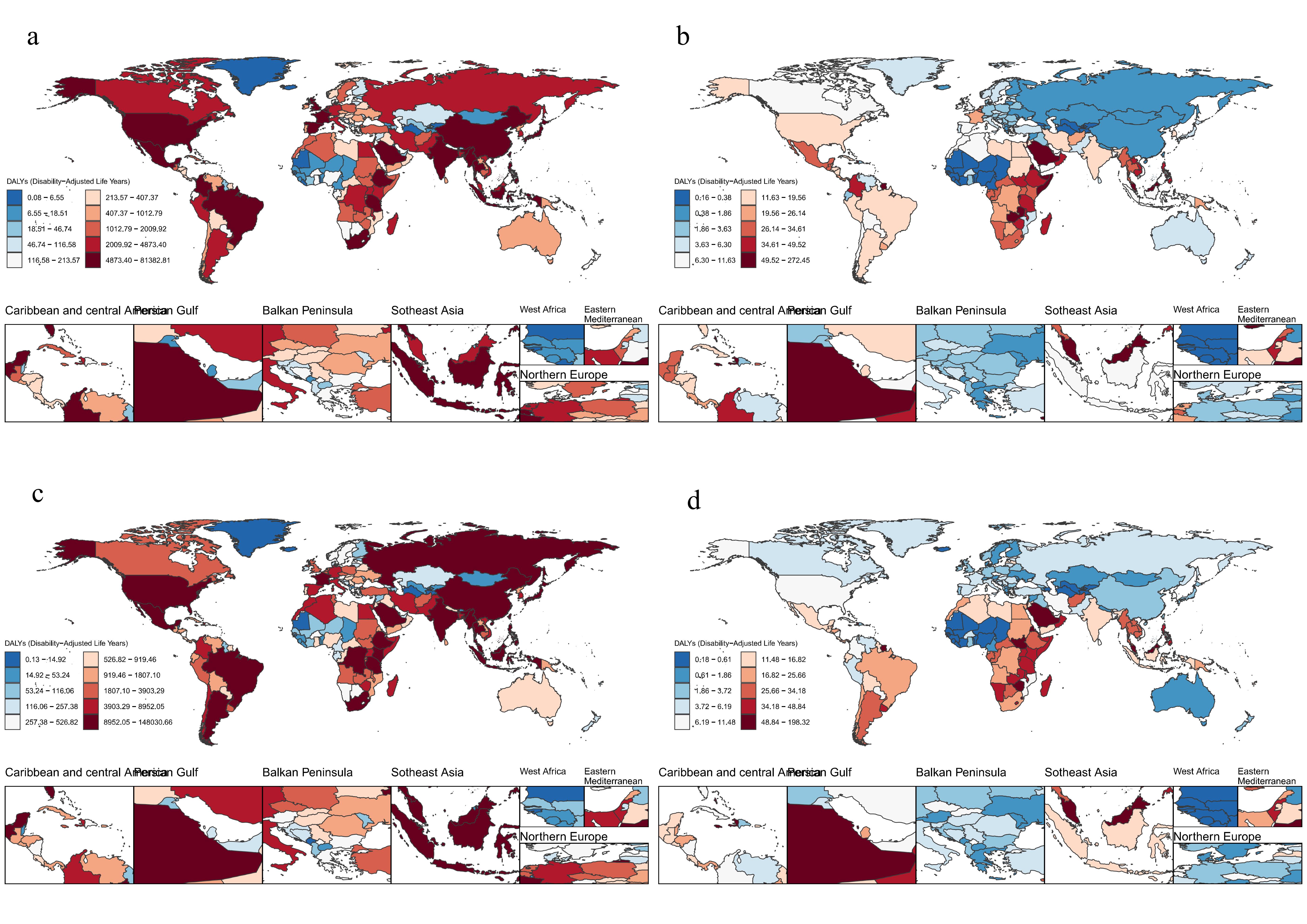

Supplement: Supplementary file 2 [file Image_2.jpeg]

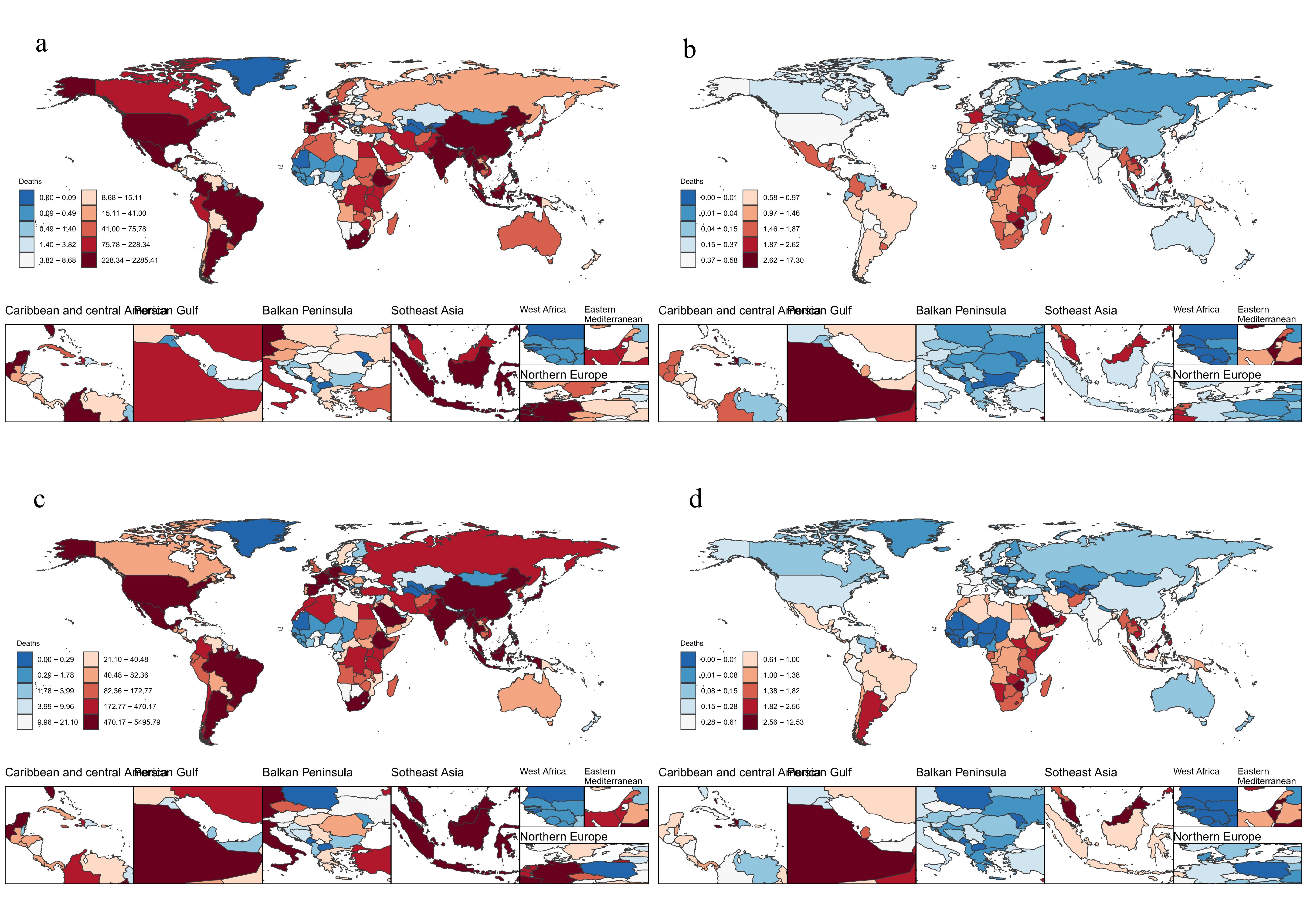

Supplement: Supplementary file 3 [file Image_3.jpeg]

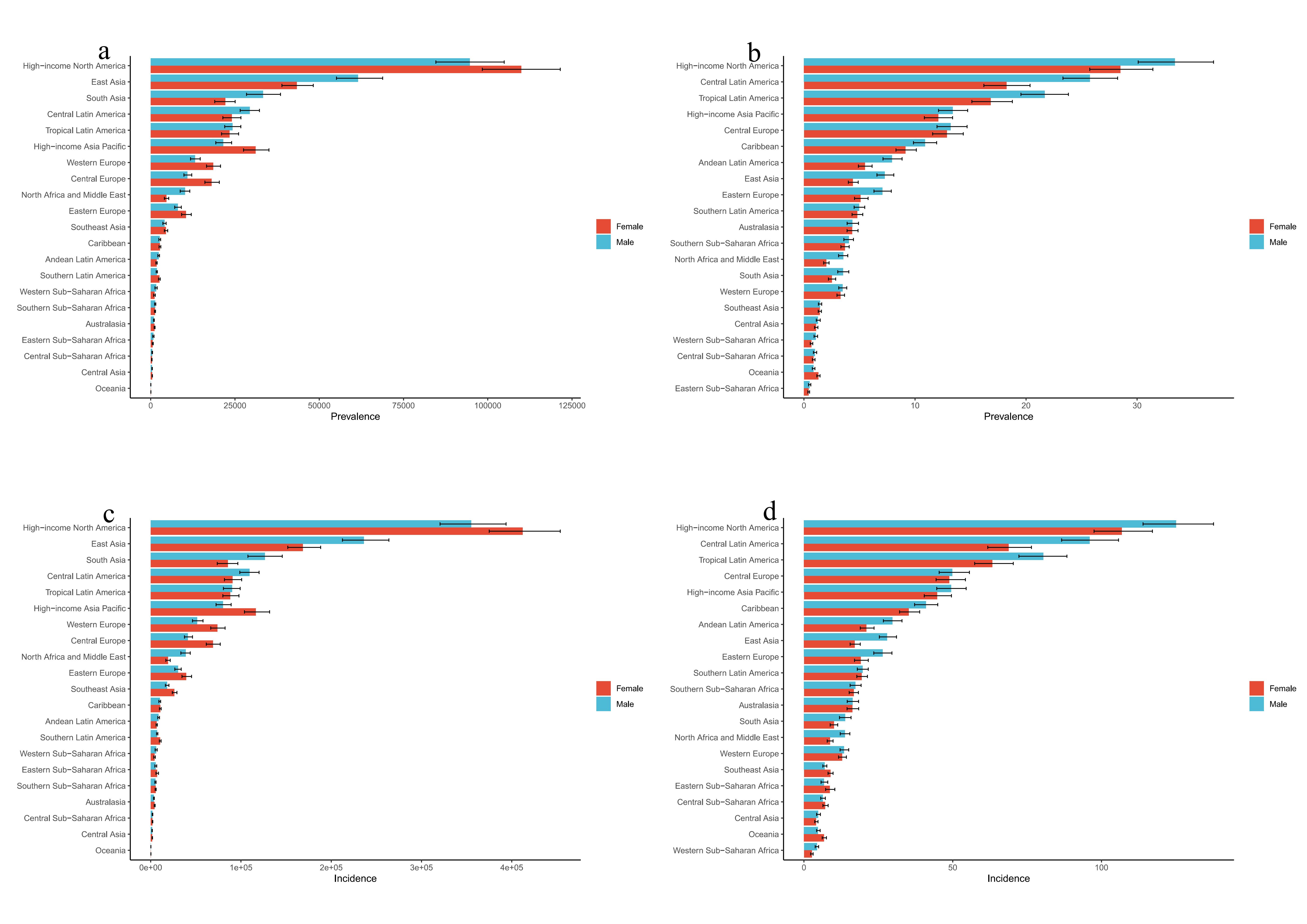

Supplement: Supplementary file 4 [file Image_4.jpeg]

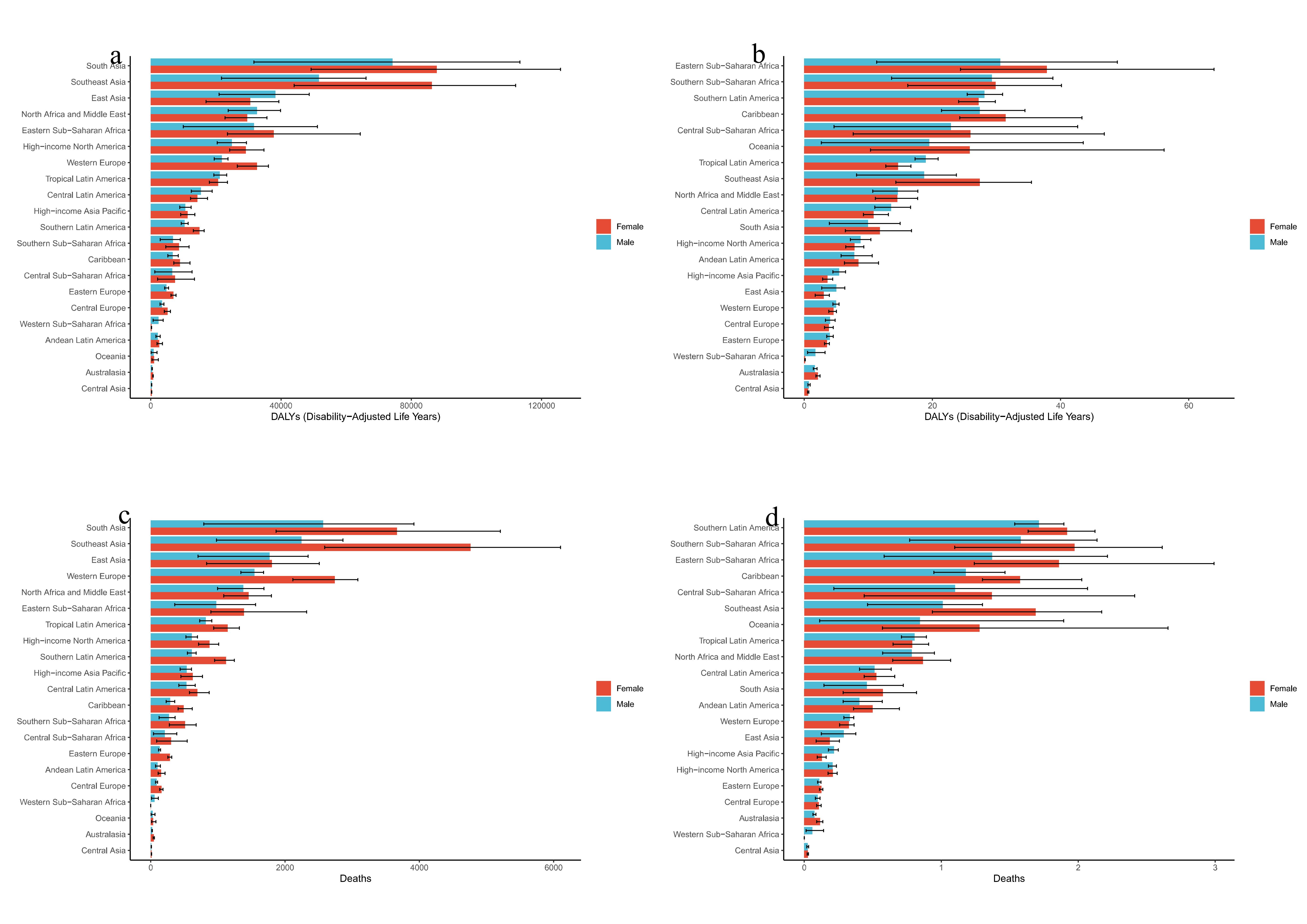

Supplement: Supplementary file 5 [file Image_5.jpeg]

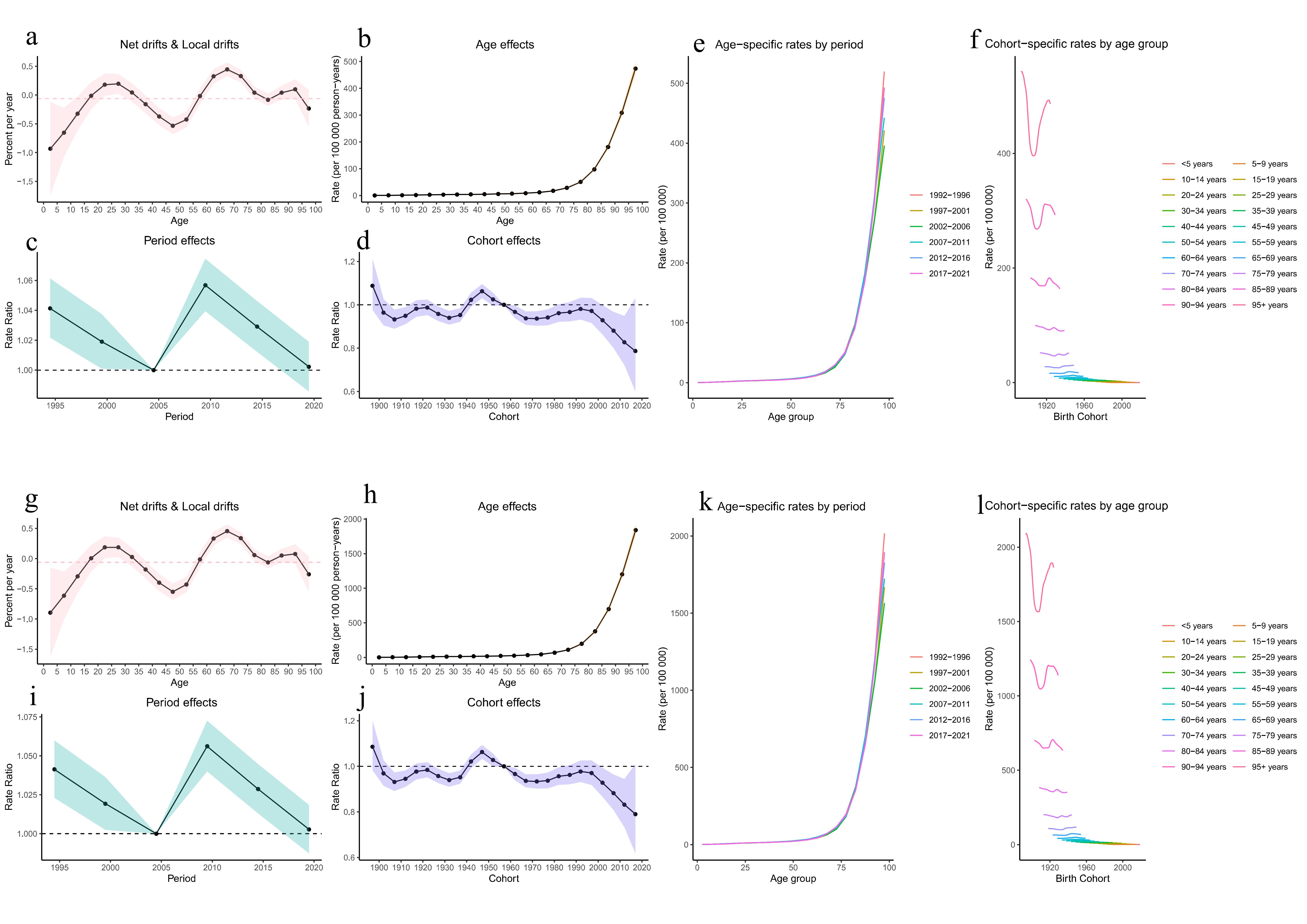

Supplement: Supplementary file 6 [file Image_6.jpeg]

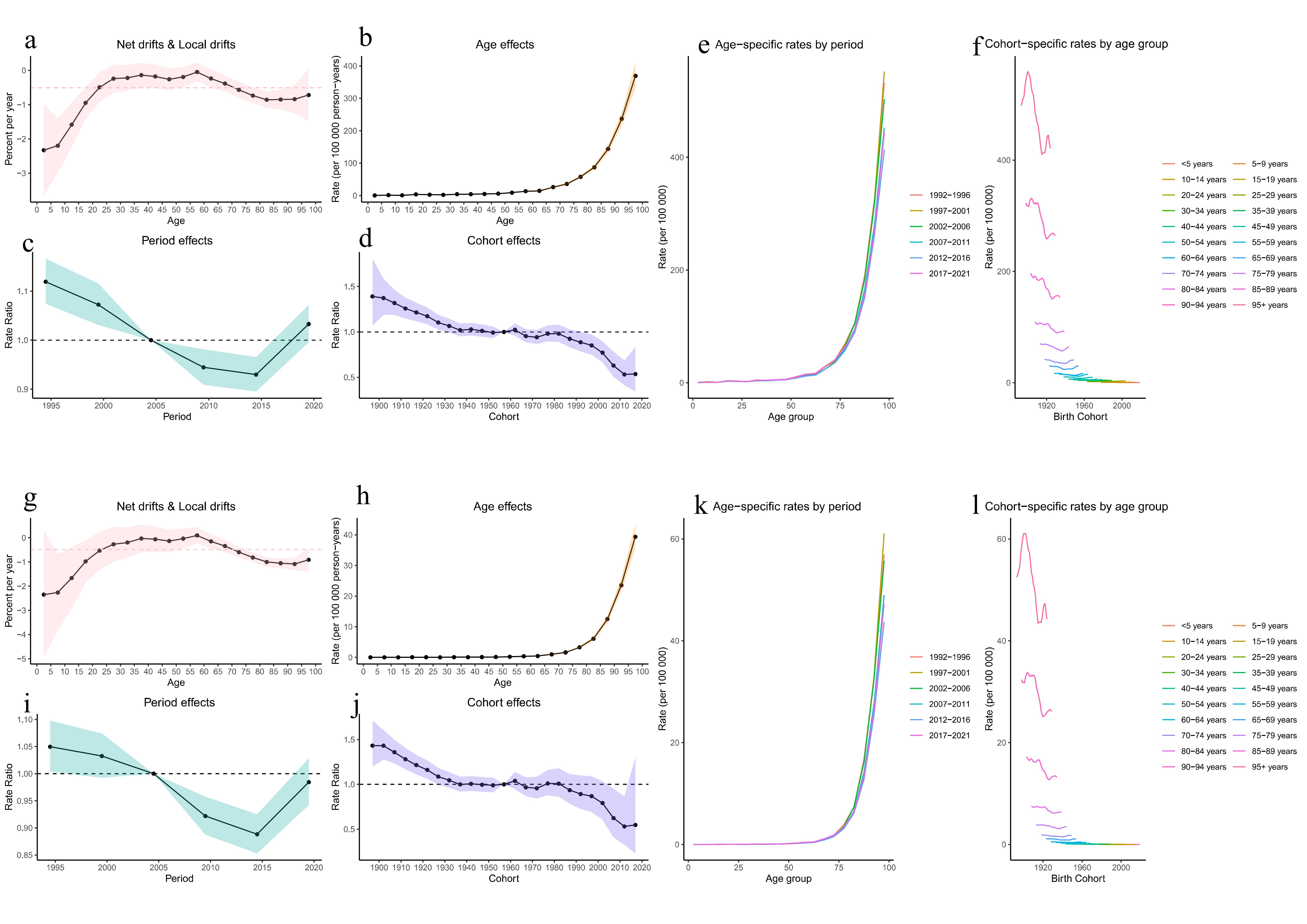

Supplement: Supplementary file 7 [file Image_7.jpeg]

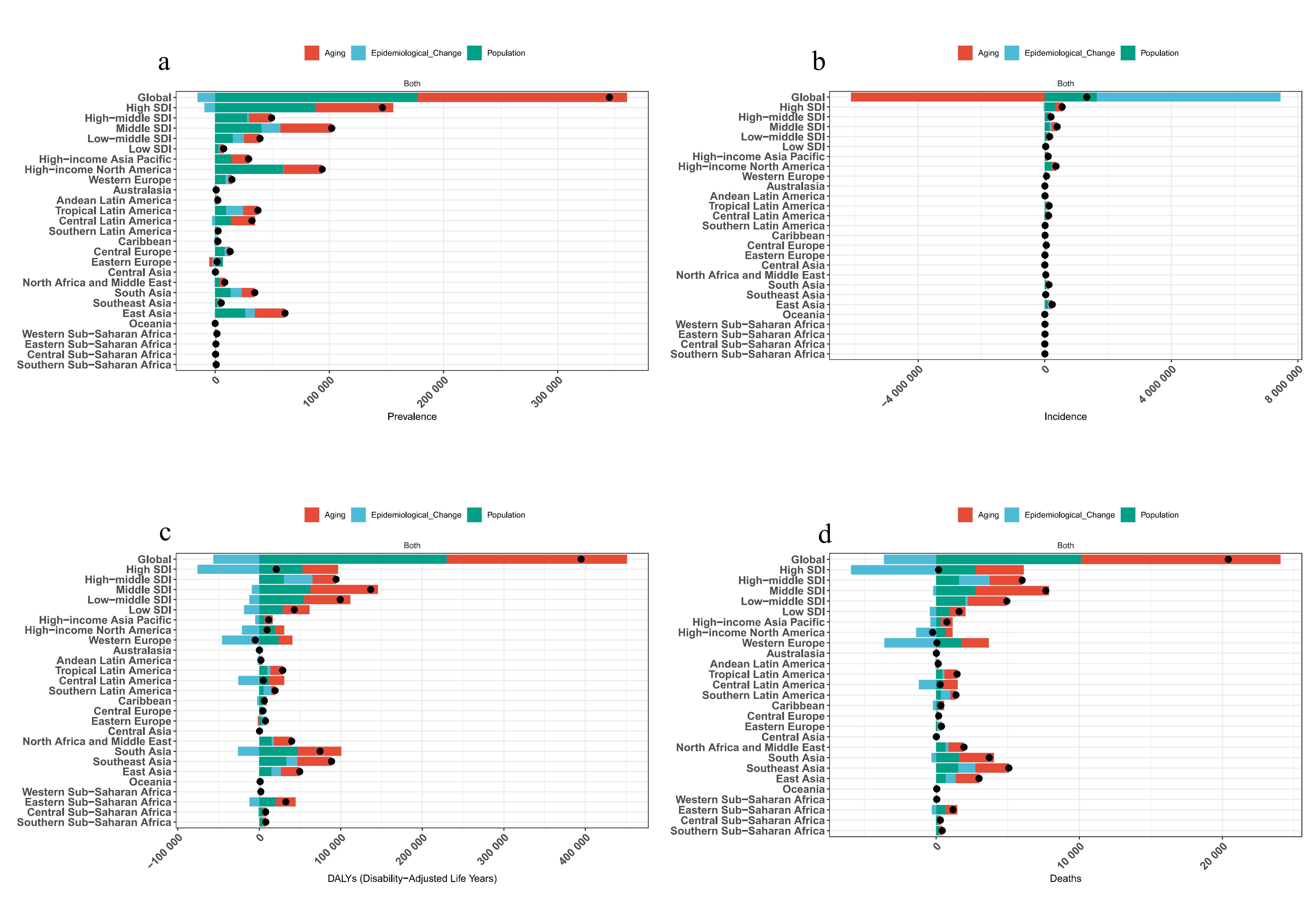

Supplement: Supplementary file 8 [file Image_8.jpeg]

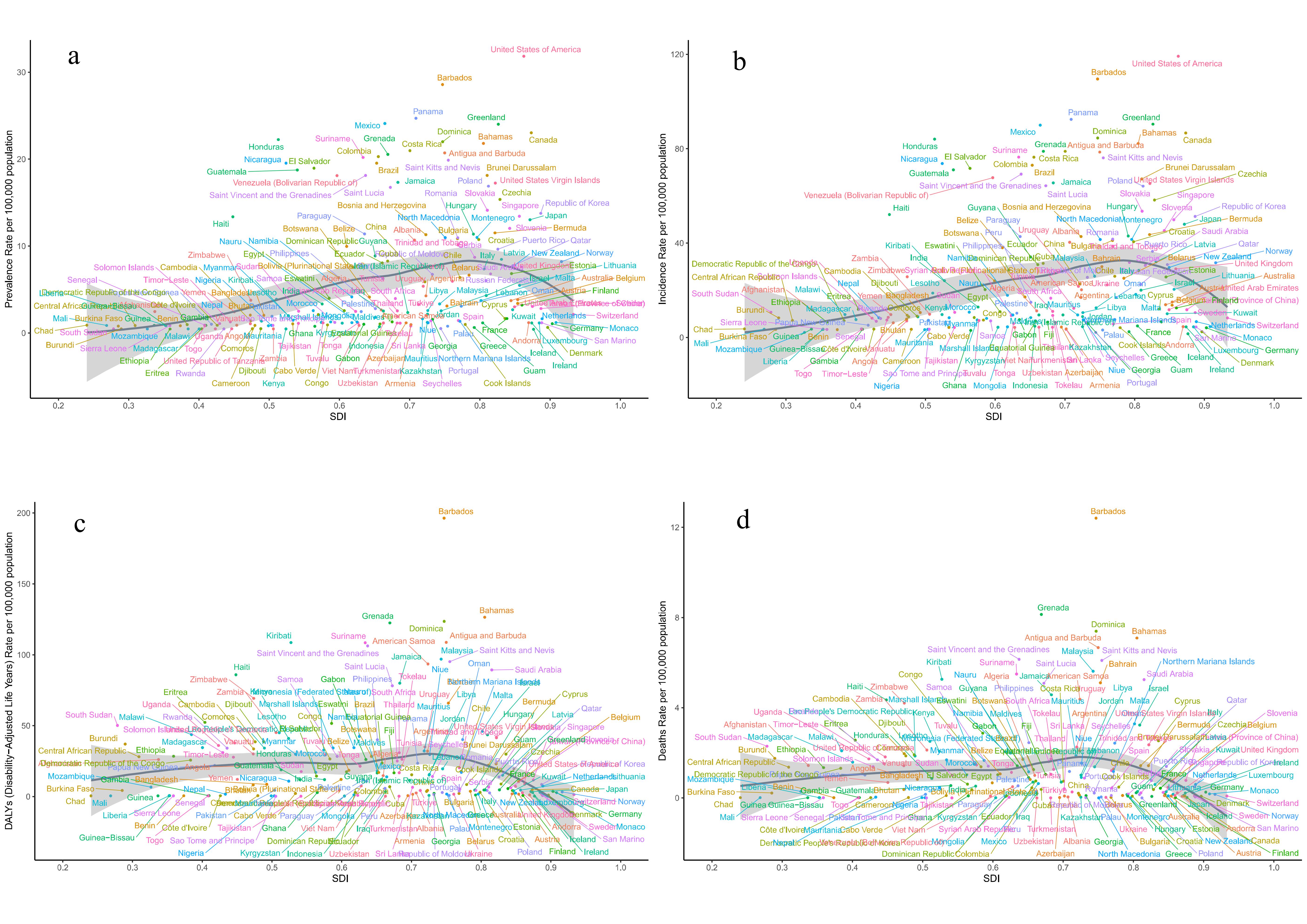

Supplement: Supplementary file 9 [file Image_9.jpeg]

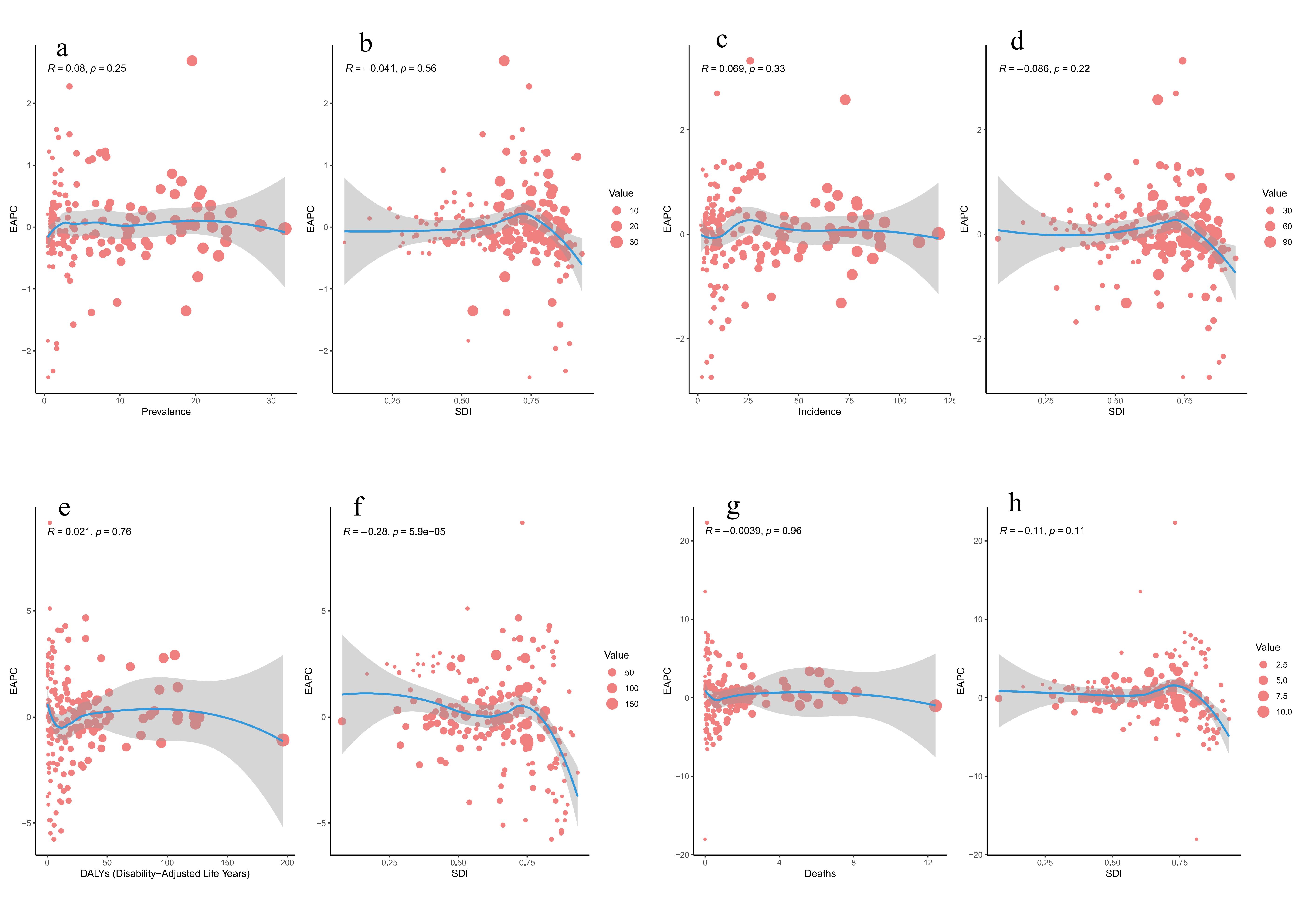

Supplement: Supplementary file 10 [file Image_10.jpeg]

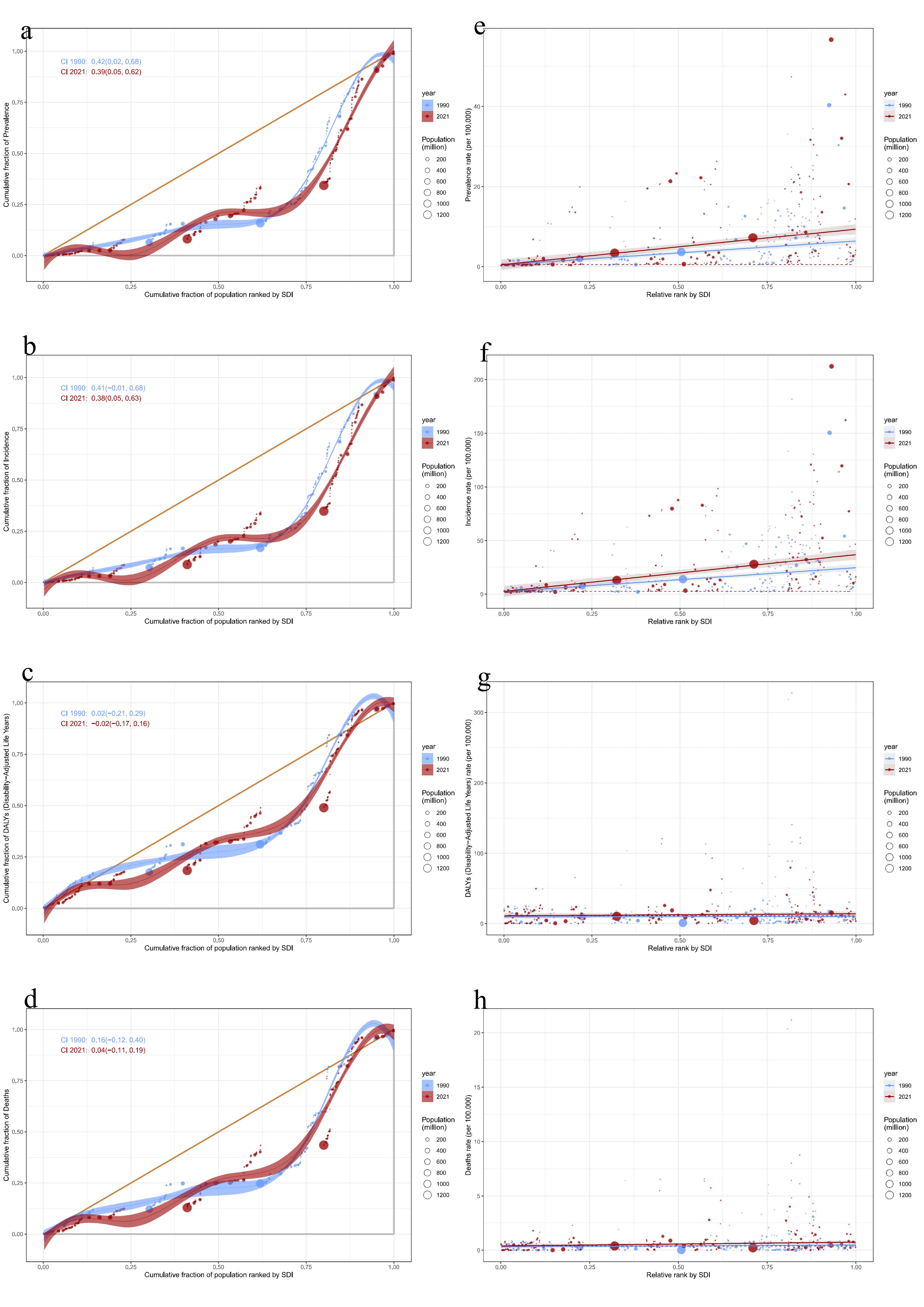

Supplement: Supplementary file 11 [file Image_11.jpeg]

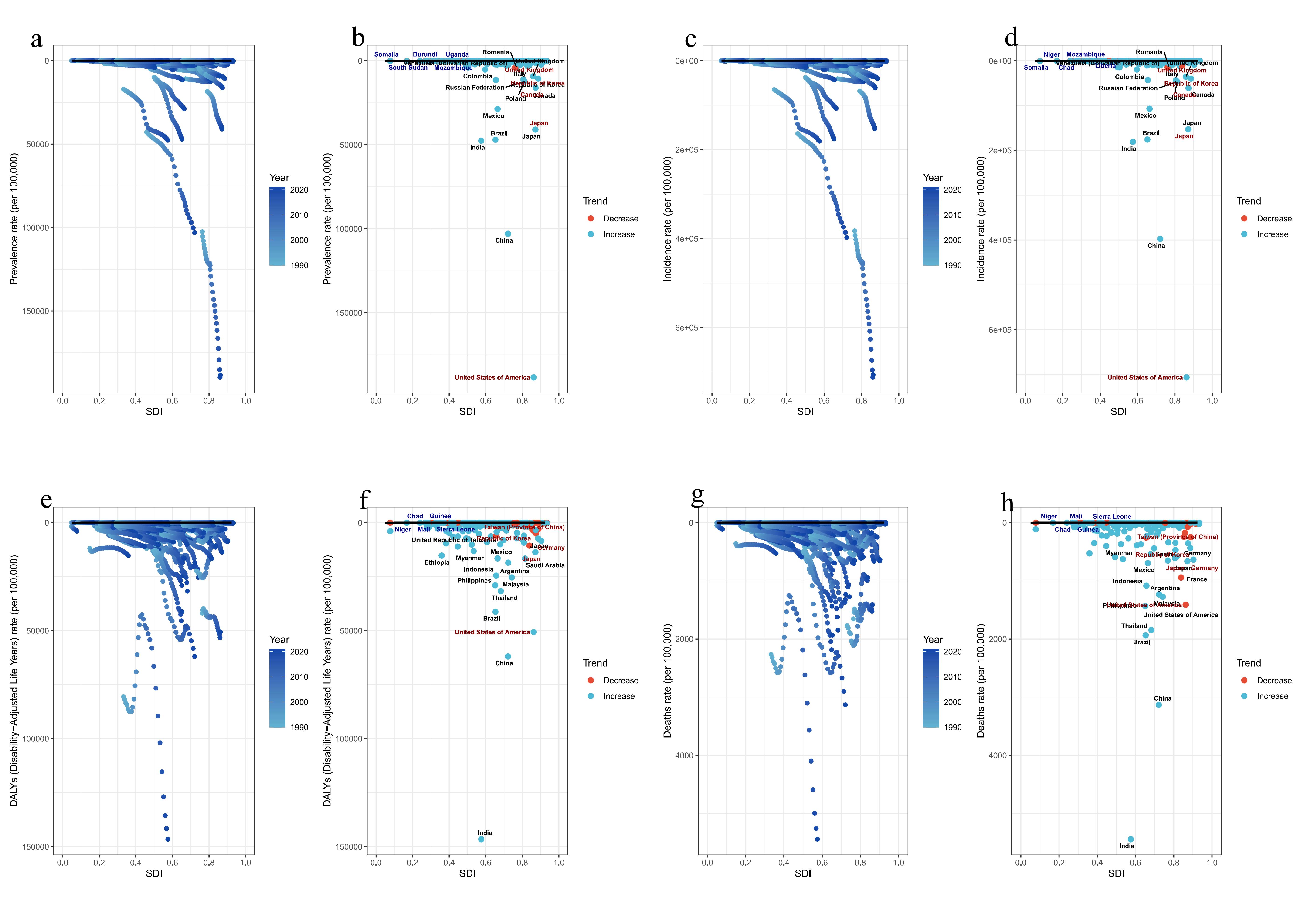

Supplement: Supplementary file 12 [file Image_12.jpeg]

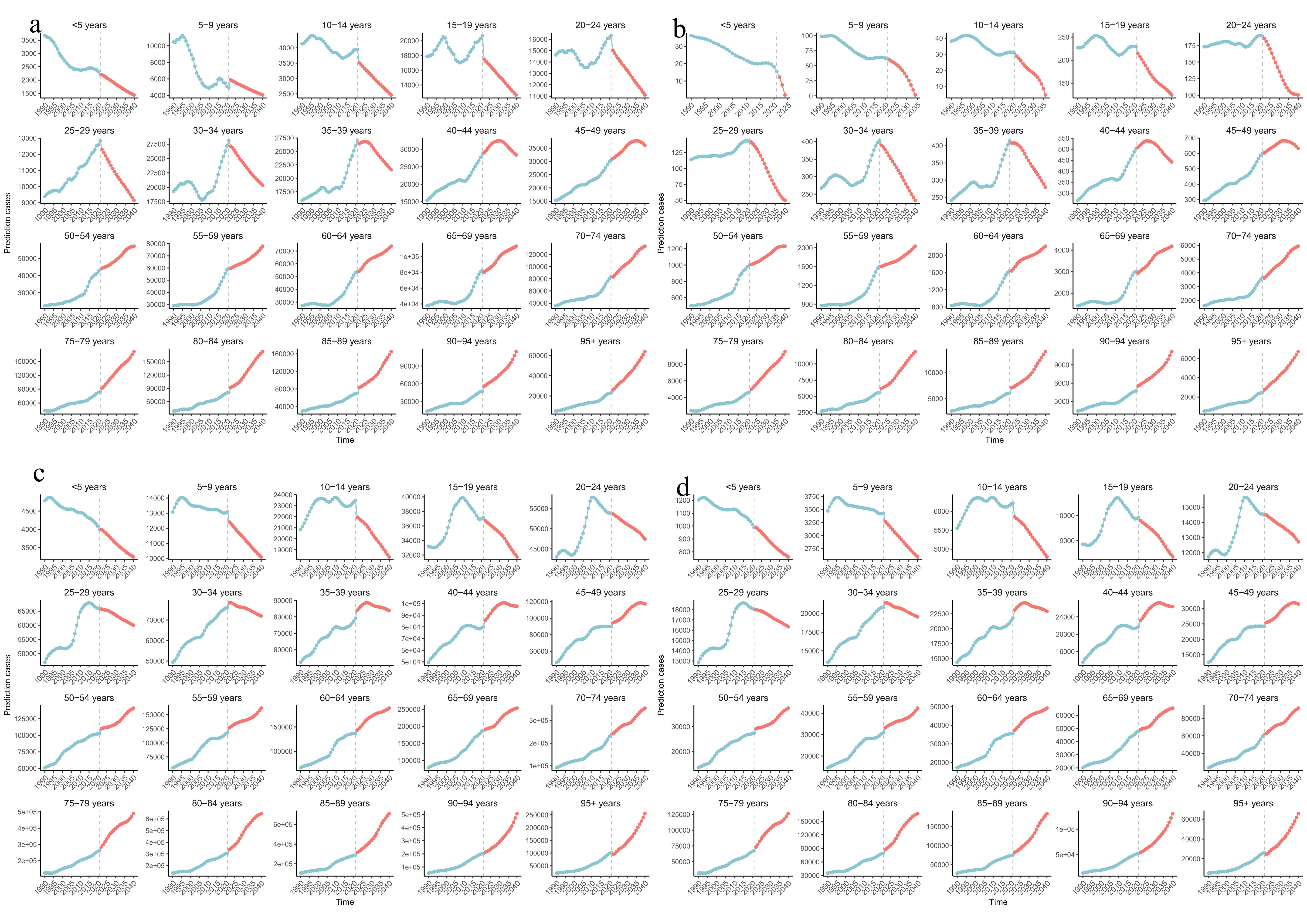

Supplement: Supplementary file 13 [file Image_13.jpeg]

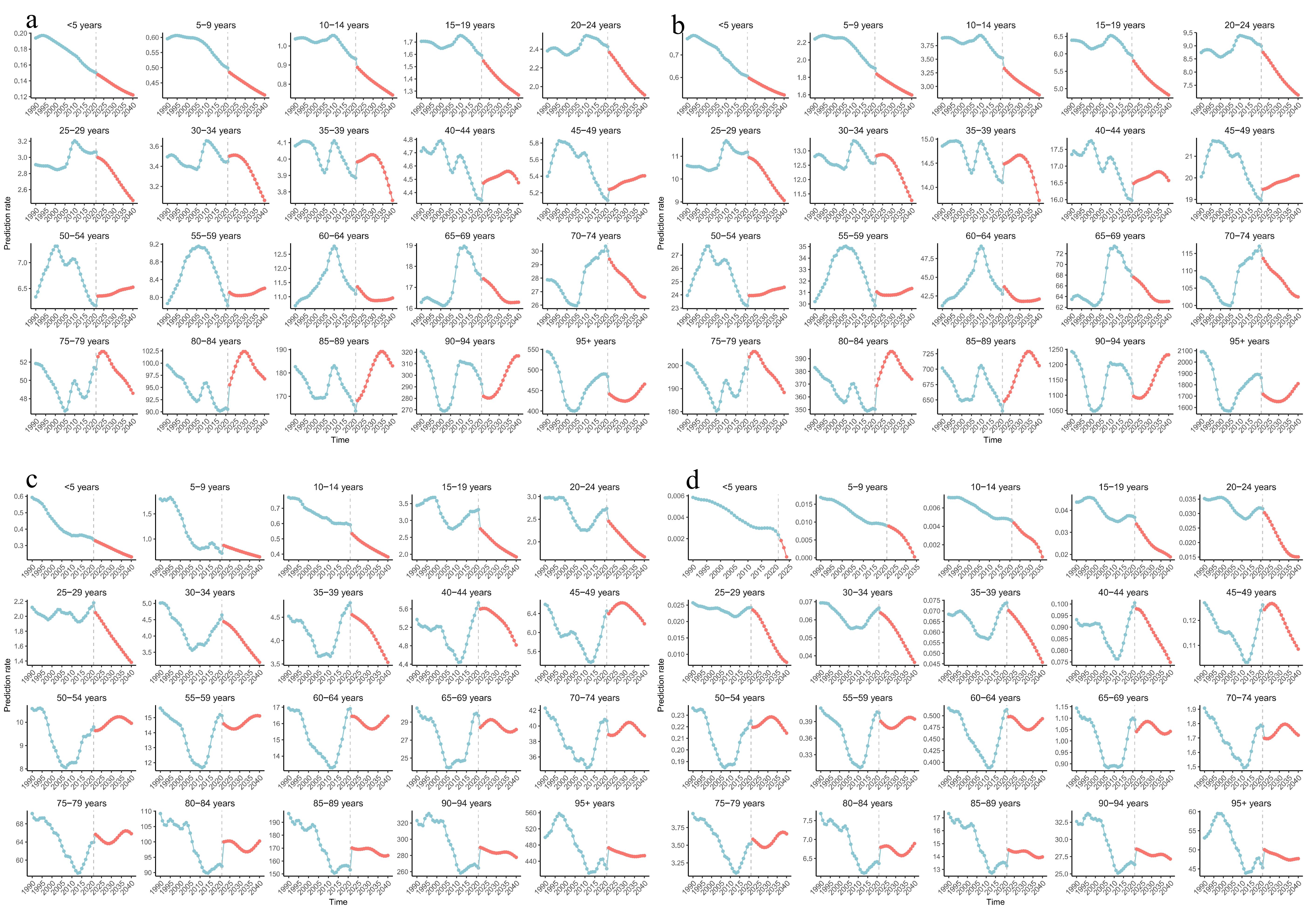

Supplement: Supplementary file 14 [file Image_14.jpeg]

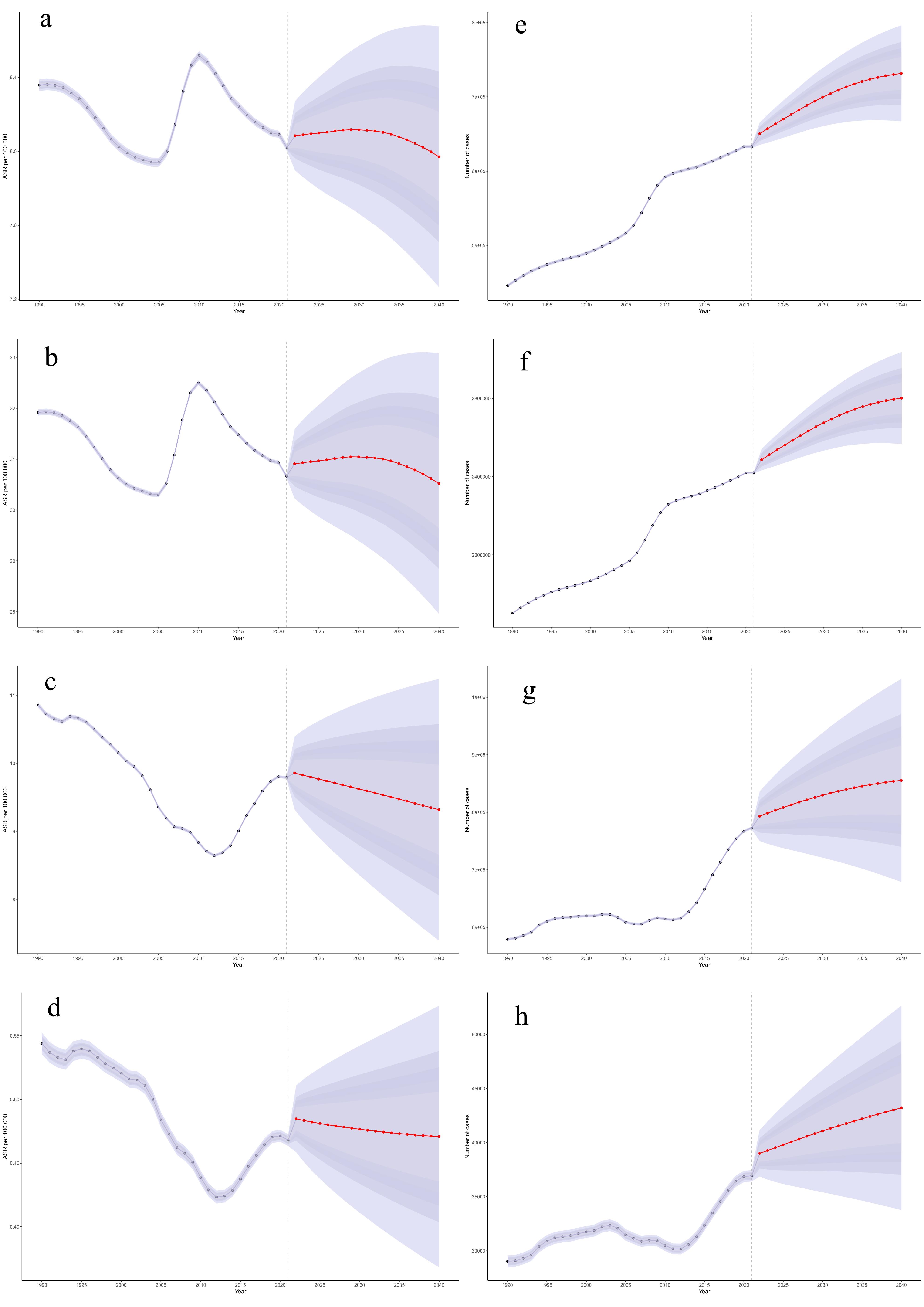

Supplement: Supplementary file 15 [file Image_15.jpeg]
